# Functional Overlay Model of Persistent Post-Concussion Syndrome

**DOI:** 10.3390/brainsci13071028

**Published:** 2023-07-04

**Authors:** Ioannis Mavroudis, Simela Chatzikonstantinou, Foivos Petridis, Octavian Dragos Palade, Alin Ciobica, Ioana-Miruna Balmus

**Affiliations:** 1 Department of Neuroscience, Leeds Teaching Hospitals, Leeds LS2 9JT, UK; 2 Faculty of Medicine, Leeds University, Leeds LS2 9JT, UK; 3 Third Department of Neurology, Aristotle University of Thessaloniki, 541 24 Thessaloniki, Greece; 4 Surgical Department, Faculty of Medicine, University of Medicine and Pharmacy “Grigore T. Popa”, 700115 Iasi, Romania; 5 Department of Biology, Faculty of Biology, Alexandru Ioan Cuza University of Iasi, 20th Carol I Avenue, 700506 Iași, Romania; 6 Centre of Biomedical Research, Romanian Academy, B dul Carol I, No. 8, 700506 Iasi, Romania; 7 Academy of Romanian Scientists, Splaiul Independentei nr. 54, Sector 5, 050094 Bucuresti, Romania; 8 Department of Exact Sciences and Natural Sciences, Institute of Interdisciplinary Research, “Alexandru Ioan Cuza” University of Iasi, Alexandru Lapusneanu Street, No. 26, 700057 Iasi, Romania

**Keywords:** post-concussion syndrome, functional neurologic disorders, mild traumatic brain injury

## Abstract

Persistent post-concussion syndrome (PPCS) is a complex and debilitating condition that can develop after head concussions or mild traumatic brain injury (mTBI). PPCS is characterized by a wide range of symptoms, including headaches, dizziness, fatigue, cognitive deficits, and emotional changes, that can persist for months or even years after the initial injury. Despite extensive research, the underlying mechanisms of PPCS are still poorly understood; furthermore, there are limited resources to predict PPCS development in mTBI patients and no established treatment. Similar to PPCS, the etiology and pathogenesis of functional neurological disorders (FNDs) are not clear neither fully described. Nonspecific multifactorial interactions that were also seen in PPCS have been identified as possible predispositions for FND onset and progression. Thus, we aimed to describe a functional overlay model of PPCS that emphasizes the interplay between functional and structural factors in the development and perpetuation of PPCS symptoms. Our model suggests that the initial brain injury triggers a cascade of physiological and psychological processes that disrupt the normal functioning of the brain leading to persistent symptoms. This disruption can be compounded by pre-existing factors, such as genetics, prior injury, and psychological distress, which can increase the vulnerability to PPCS. Moreover, specific interventions, such as cognitive behavioral therapy, neurofeedback, and physical exercise can target the PPCS treatment approach. Thus, the functional overlay model of PPCS provides a new framework for understanding the complex nature of this condition and for developing more effective treatments. By identifying and targeting specific functional factors that contribute to PPCS symptoms, clinicians and researchers can improve the diagnosis, management, and ultimately, outcomes of patients with this condition.

## 1. Introduction

Post-concussion syndrome (PCS) is a sequela of traumatic brain injury (TBI), clinically characterized by complex symptoms that includes headache, dizziness, neuropsychiatric symptoms, and cognitive impairment [1]. Although the term “post-concussion syndrome” has been used since 1934 [2], it is more appropriate to use the term “post-TBI syndrome”, as it may also occur after moderate and severe TBI or even sub-concussive head impacts [1]. There is controversy regarding PCS when its symptoms are exhibited for longer than usual [3]. Persistent PCS is characterized by faint and subjective symptoms and having undefined underlying pathophysiology, thus not necessarily suggesting long-term effects of head trauma. Additionally, its common occurrence in the general population makes PCS easily missed by the clinicians. Moreover, as the standard procedures of PCS assessment could reveal abnormal results, they do not follow consistently defined patterns. For instance, despite the fact that the cognitive symptoms of TBI are prominent, the patient’s standard assessment for chronic PCS often fails to identify any cognitive deficits [4]. However, some studies pointed out repeated concussions as a decisive predisposing factor for chronic traumatic encephalopathy, which is characterized by significant cognitive decline and neurodegenerative state [5,6]. Furthermore, as the affected population is heterogeneous and exhibits varying degrees of injury to the head and brain, it seems that the individual characteristics of the patients may alter the expression of the injury [1].

The incidence of PCS in patients with mild TBI varies widely, with reported rates ranging from 30% to 80% of these cases [7], possibly due to the differences in populations and diagnostic criteria. Several studies have attempted to establish a correlation between the severity of brain injury and the risk of PCS, using the Glasgow Coma Scale (GCS), duration of loss of consciousness or post-traumatic amnesia, and presence of brain imaging abnormalities [8,9,10,11,12]. However, the severity of the initial injury does not appear to be a reliable predictor of PCS risk. On the contrary, a history of prior concussions, especially if recent or multiple, may be a risk factor for persistent symptoms after concussion [13].

Functional neurological disorders (FNDs), also known as conversion disorders, are characterized by limb weakness, abnormal movements, or nonepileptic seizures that a neurological disease cannot explain, yet are experienced as genuine and cause distress and/or psychosocial impairment [14]. FNDs are commonly encountered in clinical settings and are associated with poor prognosis [15,16,17,18]. The estimated incidence of conversion disorders in the general population across various geographic regions is 4–12 per 100,000 individuals per year. Additionally, the community prevalence of conversion disorders based on case registries was found to be of 50 per 100,000 individuals per year [19]. In clinical settings, the point prevalence of conversion symptoms ranges from 2% to 6%. In a series of 157 patients hospitalized for Internal Medicine care, conversion disorder was diagnosed in 2% of the cases [20], while 6% of 3781 patients admitted for neurology care had it [15]. Similarly, when a neurologist evaluated 7836 outpatients, approximately 4% of them were diagnosed with conversion disorder [21].

## 2. Mechanisms and Predisposing Factors for Persistent Post-Concussion Syndrome

The distinction between persistent PCS and other medical and psychiatric disorders is vital when considering the treatment approaches that vary from one disorder to another. Similarly, the prognosis of their outcomes could vary despite the overlap of some common symptoms. In some cases, differential diagnosis can be scarce due to symptom overlap and their non-specificity. Furthermore, many other factors could contribute to the persistence of PCS symptoms. Female sex and increasing age are risk factors for PCS in patients with mild and moderate TBI [9,10,22,23].

While the nature of the head injury has not been systematically studied as a risk factor, some evidence has suggested that patients with sports-related concussions may have better prognosis than those with TBIs resulting from motor vehicle accidents, falls, or assaults [24] due to severity differences, as well as due to the physical and psychosocial impact of the injury, the differences in premorbid predisposition to PCS, and psychological factors. The relative preponderance of accidents and assaults as causes of TBI may also contribute to the observed sex differences in PCS risk. In this context, it was suggested that increased neck strength could prevent severe concussion effects [25,26]. However, a recent study failed to demonstrate that sex could influence the response to simulated neck movement associated with TBI [27], despite females generally having lower neck muscle strength as compared to males [28], while another study [26] pointed out that there could be some differences during the anticipatory activation of cervical muscles.

The prevalence of persistent PCS is difficult to determine because of the variability in the definition and the criteria used to diagnose. In some studies, the incidence ranged from 5% to 15% [2,3,4], while, in others, it could be as high as 58% [8]. A recent systematic review of studies reporting persistent symptoms of PCS in adults found prevalence estimates ranging from 7% to 58%, with the overall pooled prevalence being 32% (95% CI: 24–41%) [9].

The pathogenesis of persistent PCS is unclear. Nevertheless, it is believed to be a complex interaction of structural and functional brain changes, genetic predisposition, psychosocial factors, and healthcare solutions [11]. Studies have suggested that the pathophysiology of persistent PCS may differ from that of acute and subacute PCS [11,12,13]. Functional neuroimaging (single-photon emission computed tomography [SPECT], positron emission tomography [PET], and functional magnetic resonance imagining [MRI]) have documented persistent areas of decreased blood flow, decreased glucose metabolism, and decreased functional activity, particularly in the prefrontal and limbic regions of the brain [14,23,24,29,30,31]. These findings may represent a chronic state of decreased activity or chronic compensatory efforts and may have implications for cognitive, affective, and behavioral function [32,33]. However, these findings are not specific to PCS and may also be found in other medical and psychiatric disorders.

A psychogenic contribution to persistent PCS is suggested by the high prevalence of comorbid depression, anxiety, and post-traumatic stress disorder (PTSD) in patients with persistent PCS [34,35,36]. Psychological factors may contribute to the persistence of symptoms through negative beliefs about recovery, increased vigilance for symptoms, illness behavior, and a focus on physical rather than emotional or cognitive symptoms [37,38].

Sociocultural factors, such as litigation, compensation, and social support may also contribute to the persistence of PCS symptoms [39,40,41,42]. Patients with financial compensation claims, particularly those involving litigation, are more likely to have persistent symptoms [43,44]. However, it is unclear whether the litigation causes the persistence of symptoms or whether patients with persistent symptoms are more likely to pursue litigation [45].

Other factors that may contribute to the persistence of PCS symptoms include a history of prior head injury, genetic factors, female sex, older age, and the severity and type of the initial injury [46,47,48]. Patients with persistent PCS are more likely to have had a prior head injury, and the presence of a prior injury may be a risk factor for persistent symptoms after subsequent head injury [49,50]. However, some of the risk factors that were already reported overlap with some physiological traits, such as sex-associated muscle strength and endocrine regulation. In this context, it was suggested that females generally experiment headaches more frequently than men. Thus, the fact that female sex is reported as a risk factor for developing persistent PCS symptoms could be associated with sexual endocrine monthly regulation or muscular particularities and may not contribute to the similarity between persistent PCS and FNDs. However, several recent studies found that mTBI could lead to pituitary dysfunctions [51,52,53,54] that could further increase the risk for developing persistent headaches [55]. In this context, further studies should aim to study the implication of these aspects in diagnosis of persistent PCS [56].

Meanwhile, the persistence of PCS symptoms is associated with significant impairment in quality of life and functional status and has significant economic and social costs [57]. Management of patients with persistent PCS requires a multidisciplinary approach that addresses the complex interplay of biological, psychological, and sociocultural factors.

## 3. Mechanisms and Predisposing Factors for Functional Neurological Disorder

Despite the etiology and pathogenesis of FND remaining unclear, biological, psychological, and social factors have been identified as probable predisposing factors of FND, triggering and/or perpetuating symptoms [58,59]. Raynor and Baslet described the historical understanding over the FND etiologies and highlighted that these heterogenic premises, as well as the variable response to treatments, could suggest that FNDs are in fact complex disorders of which clear etiology could be more related to the individual than to a pathological redundant pattern [60]. In some cases, as the diagnosis of FND could be the subject of inconsistencies and discrimination [61], efforts are currently made to diminish them. In this way, Mark [62] suggested that a major source of unfavorable response (in the patient–provider relationship) could be historical biases, while McLoughlin et al. [61] pointed out that FND could be considered as a feminist issue fueled not only by historical aspects, but also by contemporary perspectives.

Psychological factors, including physical or psychological trauma, interpersonal conflicts, and recent or past stressors, may be associated with the onset of FND. These factors are not always reported or specific to the disorder [63,64]. A meta-analysis of 34 retrospective studies found that stressful life events and maltreatment were more common in patients with FND than in controls. However, 13 of these studies reported that some patients with conversion disorder did not have a history of either stressful life events or maltreatment [65]. Additionally, patients with conversion disorder are more likely to have pre-existing psychiatric disorders, other somatic symptoms, or other functional somatic disorders, such as irritable bowel syndrome, compared with controls with recognizable diseases [66,67]. Neurological illnesses, such as migraine, peripheral nerve pathology, or stroke, may also trigger but not explain the conversion symptoms, and physical injury may precede conversion symptoms [68,69,70,71]. Additionally, beliefs that there is an irreversible neurological disease, unnecessary medical investigations, inappropriate prescriptions or procedures, and disability-related financial benefits or litigation may perpetuate conversion symptoms [59,72,73,74,75,76,77]. Physical deconditioning, comorbid psychiatric disorders, and life stress may also contribute to the perpetuation of symptoms [59,72,73]. Although traditional etiological understanding of FND relied simply on the psychodynamic explanation of a physical manifestation of psychological distress as the cause of the disorder, recent etiological models have acknowledged the heterogeneity of patients with FND.

Several cognitive and neurobiological etiological models have been proposed for medically unexplained symptoms and FND. Brown and Reuber recently proposed a model that provides an integrated behavioral and psychological etiological explanation [58,59]. They used Brown’s cognitive model of unexplained illness to explain the possible sources of misleading interpretation of physical symptoms, which can be obtained through personal experience, the observation of others’ experiences or sociocultural influence about health. On the other hand, the neurobiological model of Voon and colleagues explains that FND could be characterized by conversion disorder symptoms onset coupled with increased amygdala-driven emotional arousal based on previous physical or motor experiences [64]. They suggested that the resilient processes could not inhibit the “previously mapped conversion motor representations” due to abnormal functional connectivity between the limbic structures and the supplementary motor area, as well as due to increased right amygdala, left anterior insula, and bilateral posterior cingulate area activities. In another neurobiological model for FND, Edwards and colleagues proposed a Bayesian account for FND. They suggested that functional symptoms are the result of actions based on inferences. These inferences are mediated by expectations about symptoms, past emotions, and illnesses [63].

## 4. Beliefs and Expectations in Post-Concussion Syndrome

Increasing evidence indicates that PCS symptom reporting could be influenced by non-head injury factors, suggesting that symptoms typically associated with PCS may not be unique to head injury. The role of symptom expectation in PCS symptom etiology has been hypothesized, highlighting the need to compare expected symptoms for various disorders. A study of 82 undergraduates who reported their current and expected symptoms if they had suffered a head injury, an orthopedic injury, post-traumatic stress, or depression showed no significant differences in overall symptoms or symptom subscales. However, individuals simulating head injury, post-traumatic stress, and depression expected an increase in total symptoms, whereas individuals portraying orthopedic injury did not. Individuals with head injuries reported fewer affective symptoms than those portraying psychological disorders. These results indicate that illness beliefs and expectations may play a crucial role in PCS and, more specifically, its persistence [78].

Furthermore, there are several studies that showed that some PCS patients tend to exaggerate their symptoms and their persistence due to various causes. This phenomenon was seen in both children and adults [79,80]. In order to overcome this possible limitation in the diagnosis of persistent PCS, efforts were made to include the caregivers’ observations regarding the patients’ symptoms [81]. In other circumstances, during the brain function assessment, electroencephalogram-based evaluation of left frontal neural responses during memory recollection could also bring relevant information about the possibly exaggerated symptomatology [82]. Certain scales addressing cognitive and physiological measurable aspects, such as the Mild Brain Injury Atypical Symptoms (mBIAS) scale [83,84] and the Validity-10 scale [80], could further prevent the acknowledgment of some symptoms of which severity or duration was overappreciated.

## 5. Illness Beliefs in Functional Neurological Disorders

Beliefs and expectations about health influence functional symptoms in patients with FNDs. Studies have demonstrated that patients with FNDs have a bias toward “jumping to conclusions” and frequently changing their decision when presented with new evidence, which could be a risk factor for inappropriate updating of active inference, the theory in which the brain predicts and explains sensory input through past experiences [85]. This bias is reflected in the fact that patients with FND request less information than healthy controls before forming a decision. Furthermore, patients with functional tremors have been shown to overestimate the occurrence of tremors, reporting an occurrence rate of 80–90% of their waking day. In contrast, objective measurements indicate an average occurrence of only about 30 min daily. This overestimation is significantly more significant than in patients with organic tremors, indicating that top-down prediction of constant tremors may prevent the perception of time without tremor in patients with FND. In addition, the power of symptom expectation has been demonstrated, as those who expected to experience analgesia in certain areas of their body reported analgesia in exactly those areas. This finding has been incorporated into several etiological models for general medically unexplained physical symptoms and FND. These observations suggested that beliefs and expectations play a critical role in developing and maintaining functional symptoms, and they highlight the need for further research to elucidate the underlying mechanisms of this phenomenon [86,87].

## 6. Similarities in Symptoms and Underlying Mechanisms

PPCS and FND share overlapping features, with similar symptoms and potential underlying psychological and risk factors. They both include physical symptoms such as headaches, dizziness, fatigue, and cognitive symptoms, such as memory problems and concentration problems. FND may also present with motor symptoms, such as tremors, gait abnormalities, and seizures, which can also be seen in PCS [3,4,15,16]. In addition, PPCS and FND may have underlying psychological factors contributing to symptom expression. Psychological stressors, such as trauma or emotional distress have been implicated in developing both PPCS and FND. Anxiety, depression, and PTSD are common comorbidities in PPCS and FND. There is evidence for the co-occurrence of PCS and FND. Picon et al. reported unexpected and unexplained neurological symptoms in post-concussion syndrome, which were most likely functional in nature [88]. The above suggests that there may be a relationship between these conditions, although the exact nature of this relationship is not fully understood. The similarities between PCS and FND highlight the need for careful evaluation and diagnosis and integrated treatment approaches that address the physical and psychological aspects of these conditions.

One argument for classifying PPCS as an FND is the lack of objective evidence for structural brain damage in most cases of PCS, as well as the presence of psychological or functional factors that may play a role in the expression of PCS symptoms. Furthermore, evidence suggests that psychological factors can contribute to the development and persistence of PCS symptoms. Individuals who experience high levels of stress or anxiety before or after a concussion may be more likely to develop PCS symptoms, even if the initial injury was relatively mild. This is consistent with the idea that FNDs manifest psychological distress rather than a result of structural or physiological abnormalities. One would argue that PCS shares significant similarities with FND. At the same time, both PCS and FNDs can present with a wide range of physical and cognitive symptoms that are not fully explained by underlying structural damage or other medical conditions. Additionally, treatment for PCS and FND often involves a multidisciplinary approach that addresses both physical and psychological factors. The lack of objective evidence for structural brain damage in PCS, combined with evidence of psychological factors contributing to PCS symptoms and similarities with other FNDs, suggests a strong case for considering PCS as an FND.

## 7. FND Overlay Model of PCS

Symptoms of PCS, particularly persistent ones, may be related to underlying psychological factors, such as anxiety, depression, or PTSD. These factors may contribute to the development and maintenance of PCS symptoms. One would argue that FND and PCS share many features with FND, such as the presence of physical symptoms that are not fully explained by underlying structural or physiological abnormalities and the high prevalence of comorbid psychiatric disorders, such as depression and anxiety, in individuals with PCS.

**Figure 1:**
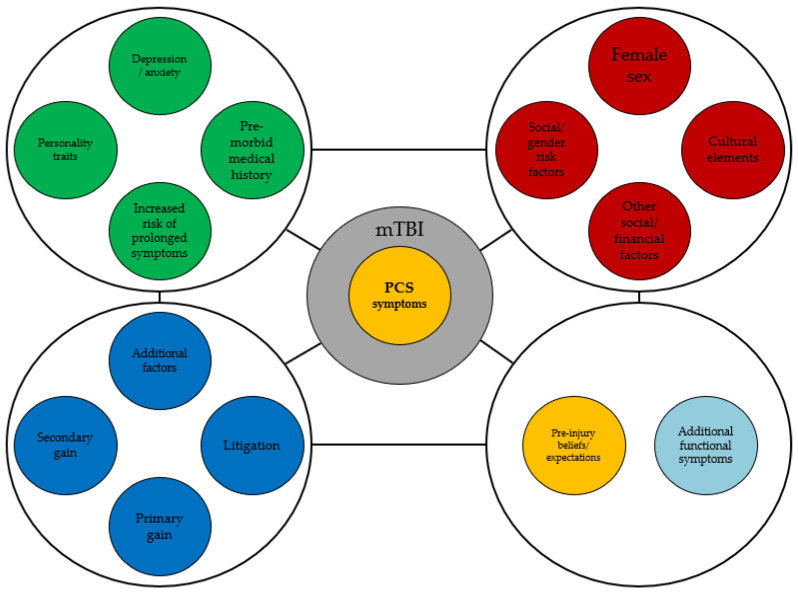
The functional overlay model offering a potential framework for conceptualizing PCS as an FND.

One potential model for conceptualizing PCS as an FND is the “functional overlay” model. This model suggests that PCS symptoms may be influenced by psychological and behavioral factors, which can exacerbate underlying neurological impairments and contribute to persistent symptoms. In this context, individuals with PCS may experience a range of neurological symptoms, such as changes in cognitive function, mood, and sensory processing, which are related to the underlying brain injury. These impairments may be exacerbated in some cases by psychological and behavioral factors, such as anxiety, depression, or maladaptive coping strategies. Thus, the psychological and behavioral factors could contribute to the development of PPCS by amplifying or maintaining neurological impairments. For example, anxiety or stress may increase physiological arousal, leading to changes in sensory processing or attentional focus. Similarly, maladaptive coping strategies, such as avoidance or overexertion, may exacerbate neurological impairments and contribute to the development of chronic symptoms. Furthermore, addressing psychological and behavioral factors may be important in treating PCS as an FND. This may involve a multidisciplinary approach, including cognitive behavioral therapy, physical therapy, and medication management. Overall, the functional overlay model provides a potential framework for conceptualizing PCS as an FND and suggests that addressing psychological and behavioral factors may be an essential component of effective treatment (Figure 1).

## 8. Limitations of FND Diagnostic Criteria

The DSM-V diagnosis criteria [14] of FND require one or more symptoms that affect body movement or senses that cannot be explained by a neurological or other medical condition or another mental health disorder, but that cause significant distress or problems in social, work, or other areas, or that are significant enough for medical evaluation to be recommended. Patients with PPCS may exhibit one or more symptoms of cognitive impairment that may cause distress or impairment of functioning. Can their presentation be attributed to another medical condition? Data failed to establish a direct link between the severity of an mTBI and the duration or the severity of cognitive symptoms. The initial symptoms could be attributed to the head impact and the physiological cascade that follows; however, persistent cognitive symptoms are mainly related to certain psychological factors, similar to the predisposing and risk factors for FND.

Furthermore, most PPCS patients with cognitive impairment describe symptom fluctuations and inconsistencies in clinical presentation. It seems that, in general, PPCS with cognitive impairment fulfils the diagnostic criteria for FND.

Despite this, there are some limitations to the diagnostic criteria for FND that may limit its utility in classifying PCS. The current diagnostic criteria for FND, or the DSM-V criteria for conversion disorder, rely heavily on excluding alternative medical explanations for symptoms. However, in the case of PCS, there is a clear medical explanation for the initial injury that may be more relevant to the patient’s symptoms than underlying psychological factors.

## 9. Critiques and Limitations of the FND Model for PPCS

The main arguments against the FND model would be that PCS is a distinct clinical entity with clear neurobiological underpinnings, and that there is no evidence to suggest that psychological factors play a primary role in the development of PCS symptoms. The FND model is too broad and lacks specificity, which may lead to overdiagnosis and overtreatment of individuals with PCS. The controversy surrounding whether PCS is an FND highlights the need for continued research to better understand the underlying mechanisms of PCS and develop effective treatments that target the condition’s physical and psychological aspects. One of the main arguments against PCS as an FND would be that it has distinct underlying neurological factors that are not present in other FNDs. Unfortunately, the pathophysiology of FNDs is still not fully understood; thus, further research would help in formulating more relevant observations regarding the overlap between PCS and FNDs. However, as previously argued by the general definition of FNDs, PCS can result from a specific traumatic event that is an mTBI, which can cause measurable changes in brain structure and function. Thus, the psychological factors may contribute to developing and maintaining PCS symptoms, but are not the primary cause.

Moreover, as stated before, the current model meets some methodological limitations that are due to the multifactorial aspects of both PCS and FNDs. In this way, it was suggested that one solid source of non-specificity could be the fact that most health impairments that include a psychological component should be regarded as an interaction between the physiological response and the psychological status. This interaction could further predispose to worsened symptoms or poorer outcomes following treatment (such as pre-existing psychiatric disorders). Thus, further research on this model should find additional evidence to support more specific shared pathophysiological aspects that could finally narrow down the focus to PCS and FNDs by explaining similarities between the two, as well as differences between PCS and other unexplained chronic neuropsychological disorders. For example, some similarities between PCS and long COVID-19 were recently reported by Davidson et al. [89], who tested the hypothesis that PCS assessment in long COVID-19 patients would provide a mechanistic framework to treat the latter. Moreover, Teodoro [90] recently found relevant evidence to diagnose FNDs in long COVID-19 patients.

On the other hand, most of the current data were obtained from the evaluation of PCS cases that were the result of sports-related and military-related concussions, along with only a few cases that were due to car accidents. In this context, further research should channel their attention to other causes in order to test the whole patient care approach in PCS versus FNDs.

Additionally, it was shown that some aspects of the holistic approach in treating FNDs could be applied in PPCS with promising results [91,92,93], and this could be a reason to suspect that they are based on similar grounds in terms of symptoms persistence mechanisms, or that the holistic approach is generally of use in multiple component neurological disorders. However, some studies reported that the provider–patient relationship could be affected by an FND diagnosis [62,94,95]; thus, further research could better describe the degree of overlap between FND and PPCS in order to find the source of similarity between them or to test the possibility that the mechanistical aspects could better suggest overlaps with other chronic neurological disorders.

Further research should also elucidate if the perception of persistent PCS patients of themselves and their illness would be a factor in influencing or evaluating the physiological and psychological outcomes following holistic treatment, as it was suggested that self-perception and expectations related to the disease could at least partly change the treatment and recovery outcomes in FNDs and other chronic neurological disorders [96,97,98].

## 10. Concluding Remarks

FNDs are characterized by neurological symptoms causing substantial distress or impairment in social, occupational, or other important areas of function, which warrant medical evaluation and cannot be explained by another medical condition, along with clinical evidence of internal inconsistency.

PCS is a sequela of mTBI. The prevalence of PCS varies and depends on pre-injury factors, patient population, assessment, analytic strategies, diagnostic criteria, and classification methods. Post-concussive symptoms (more specifically, cognitive impairment), a major symptom in PCS, also fulfill the diagnostic criteria for FND, as seen in other populations. Thus, PCS and FND share common risk and predisposing factors. Both FND and PCS can be influenced by pre-injury mental health status. Psychological factors, such as recall bias, beliefs, and expectations about health, can also influence the reporting of symptoms in both conditions. Furthermore, individuals with FND and PCS have a decreased sense of agency or control over their actions.

Despite these similarities, there are also differences between FND and PCS. Cognitive impairment in PCS has a clear pathophysiology and cause, while cognitive impairment in FND does not have a clear organic basis. In addition, there is a lack of objective evidence for cognitive impairment in FND, whereas, in PCS, neuropsychological testing consistently shows minor cognitive deficits within the first 2 weeks after injury, with some evidence suggesting deficits lasting up to 6 months. In conclusion, FND and PCS share similarities and common risk and predisposing factors, including pre-injury mental health status and psychological factors. Clinicians should be aware of these similarities and differences when evaluating patients with cognitive complaints.

## Data Availability

Not applicable.
